# Dark Mode Excitation in Three-Dimensional Interlaced Metallic Meshes

**DOI:** 10.1021/acsphotonics.0c01811

**Published:** 2021-03-03

**Authors:** Alexander W. Powell, Rhiannon C. Mitchell-Thomas, Shiyu Zhang, Darren A. Cadman, Alastair P. Hibbins, J. Roy Sambles

**Affiliations:** † Electromagnetic and Acoustic Materials Group, Department of Physics and Astronomy, University of Exeter, Exeter EX4 4QL, United Kingdom; ‡ Wolfson School of Mechanical, Electrical and Manufacturing Engineering, Loughborough University, Loughborough LE11 3T, United Kingdom

**Keywords:** metamaterials, 3D printing, longitudinal modes, microwaves, beam shaping

## Abstract

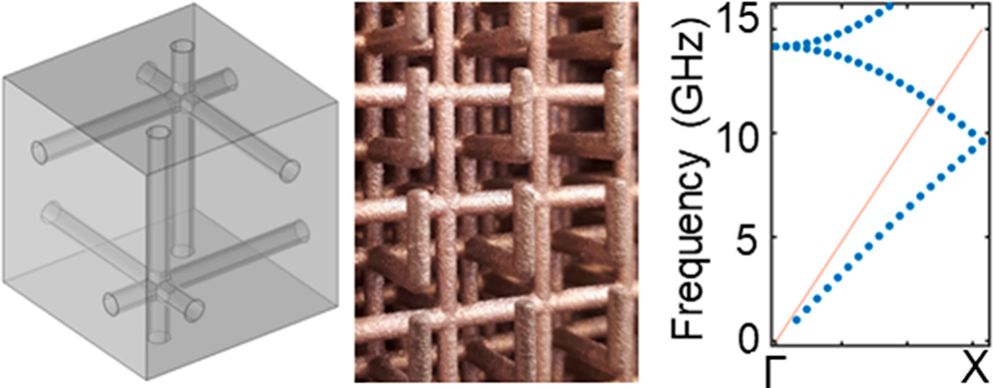

Interlaced metallic meshes form a class of three-dimensional metamaterials that exhibit nondispersive, broadband modes at low frequencies, without the low frequency cutoff typical of generic wire grid geometries. However, the experimental observation of these modes has remained an open challenge, both due to the difficulties in fabricating such complex structures and also because the broadband mode is longitudinal and does not couple to free-space radiation (dark mode). Here we report the first experimental observation of the low frequency modes in a block of interlaced meshes fabricated through 3D printing. We demonstrate how the addition of monopole antennas to opposing faces of one of the meshes enables coupling of a plane wave to the low frequency “dark mode” and use this to obtain the dispersion of the mode. In addition, we utilize orthogonal antennas on opposite faces to achieve polarization rotation as well as phase shifting of radiation passing through the structure. Our work paves the way toward further experimental study into interlaced meshes and other complex 3D metamaterials.

## Introduction

Three-dimensional structures constructed from thin metal wires have been central to the field of metamaterials since its origins. One of the earliest key discoveries in the field was that a grid or mesh of thin, electrically conductive wires behaves like a low density plasma, or dilute metal, with a plasma frequency that is tunable via the structural parameters of the grid.^1,2^ This has been fundamental for establishing negative-permittivity materials used in the experiments that have brought metamaterials into the public eye, leading to effects such as negative refraction,^3,4^ spoof plasmons,^5−7^ and cloaking.^8,9^ However, beyond some early proof-of-concept studies which only showed samples of a very limited scale,^10,11^ the difficulty of fabrication of these grids has meant that they have received little experimental attention.

Recently there has been significant interest in more complex wire mesh structures, such as the case of multiple interlocking, but unconnected meshes.^12−16^ Theoretical investigations of these structures have shown that they exhibit an unusual mode structure and do not display the low frequency cutoff characteristic of the “dilute metal” single mesh, but can support modes at arbitrarily low frequencies.^12,14,15^ Additionally, these interlaced meshes have shown the potential to behave as non-Maxwellian media, a metamaterial where the *effective* medium described in the low-frequency limit does not conform to Maxwell’s equations^12^ (despite being formed of unit cells that do) and affords new ways to control the dispersion of radiation traversing a 3D material. This unusual behavior is seen to arise from the additional degrees of freedom of the local field due to the proximity of multiple unconnected meshes with different local electric potentials.^12^ However, due to the unusual, lateral spatial modulation of the modes supported, it appears almost impossible to excite them via a plane wave. Latioui et al. showed that although this was theoretically achievable under exceptional conditions (grazing incidence and meshes with extreme difference in wire radius),^14^ such conditions are not realistic for experimental purposes. Additionally, as with the case of single meshes, it has proven difficult to fabricate and experimentally investigate these structures. However, in recent years, new advanced manufacturing techniques such as 3D printing have revolutionized what it is now possible to create, opening up the possibility of exploring these 3D interwoven structures in the laboratory.

In this study, we fabricate these structures via selective laser sintering (SLS) then metallization and resolve the issue of exciting the low frequency dark modes of an interlaced mesh with free-space radiation via the use of a monopole antenna array connected to one of the grids.

We first experimentally demonstrate the transmission of microwaves through the standard 3D single mesh and determine the dispersion of the fundamental mode. We then characterize the use of an array of monopole antennas to couple into a double interlaced mesh and utilize this technique to transmit radiation through the mesh, and experimentally determine the dispersion for the “dark mode”. Finally, we demonstrate the possibility to achieve polarization rotation and phase shifting using this antennae-coupled interlaced dark mode mesh.

## Exploring Single and Double Meshes

**Figure 1:**
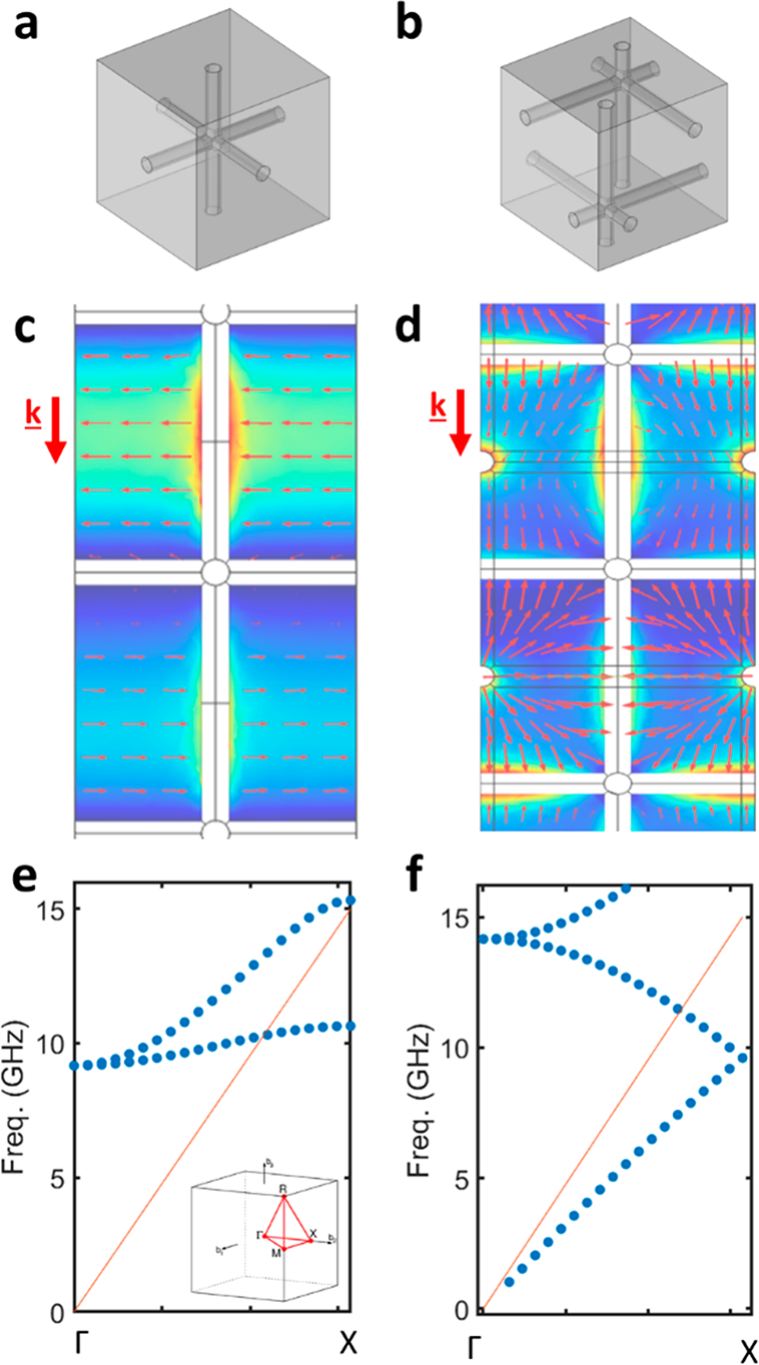
Structure of the single mesh (a) and double nested mesh (b). The electric field magnitude (colors) and direction (arrows) for the transmitted mode in the single mesh are shown in (c) and those for the low-frequency mode in the double mesh are shown in (d) for the plane parallel to the wavevector. (e, f) Dispersion relations of both meshes as simulated in Comsol for a mesh with a 1 cm lattice constant and a rod thickness of 0.5 mm with the second mesh situated exactly in the middle of the first for the double mesh structure. The inset shows the first Brillouin zone for a cubic lattice and the red line represents the light line.

The basic structures of the single and double cubic wire meshes are shown in Figure 1a and b, respectively. In Figure 1c, it can be observed that the electric field within the single mesh essentially remains polarized transverse to the wavevector, and in this respect it is like a wave in free space as shown in Figure 1e, allowing the structure to transmit effectively above the low-frequency cutoff determined by the effective plasma frequency of the grid. The double mesh is quite different, however: the polarization of the low frequency mode is quasi-longitudinal (Figure 1d), with the spatially averaged electric field largely being polarized in the same direction as the wavevector, **k**. Thus, although there are electromagnetic modes within the structure (dark modes), an external plane wave will not be able to couple to these and will be fully reflected up to the frequencies where higher order modes begin to transmit at around 14 GHz. The “dark” mode is seen in Figure 1f to lie below the light line and to be nearly dispersionless from around 10 GHz down to the DC.

## Experimental Observation of Single Mesh Modes

**Figure 2:**
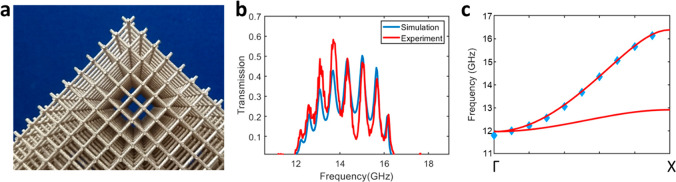
(a) Photograph of the metallized 3D printed single grid. (b) Simulated and experimental transmission for a single grid with a lattice parameter of 1 cm, a wire radius of 0.9 mm, RMS surface roughness of 0.1 mm, and conductivity of 1.85 × 10^5^ S/m. (c) Simulated and experimental dispersion relation of the grids, derived from the Fabry–Perot peaks.

It has historically been very difficult to examine 3D meshes experimentally due to the problems with fabrication, and previous studies show samples, usually made from sheets of PCB fitted together,^2,10^ and always only a few unit cells thick,^11^ allowing for limited exploration. However, with modern 3D printing technology, it is now possible to fabricate very complex structures with far fewer processing restrictions than conventional manufacturing techniques. For complex three-dimensional structures in the microwave regime, selective laser sintering techniques are especially applicable, as the powder-bed nature of the printing process removes the need for supports, allowing for significantly greater design freedom than other methods. Figure 2a shows a cubic mesh of 15 × 15 × 10, 1 cm unit cells with a wire radius of 1 mm and a lattice spacing of 10 mm fabricated from PA12 nylon in an EOS Formiga P100 SLS 3D printer. The sample was metallized by dip-coating into a MG Chemicals “Super shield” conductive paint, with a conductivity of 1.85 × 10^5^ S/m. SLS is a method that does not leave a perfectly smooth surface, and these samples were left with an RMS surface roughness of 0.1 mm. The transmission of such a structure, as modeled on COMSOL multiphysics and measured experimentally, is plotted in Figure 2b. Experimental data was taken by placing the sample between two horn antennas and measuring the transmitted signal on an Anritsu 20 GHz VNA instrument. The sample is placed 3 m from the emitting antenna to ensure that all radiation reaching the sample is planar with approximately the same wavevector. We observe a series of peaks within an envelope corresponding to the first pass band of the structure as shown in Figure 1e. The peaks are the Fabry–Perot resonances formed from reflections at the faces of this finite lattice. These peaks fall below the unity value expected for a perfect conductor due to the finite conductivity of the paint and the roughness of the prints. Excellent agreement between the simulated and measured transmission is observed, although there is some broadening of the measured peaks, which can be attributed to small variations in the wire dimensions from the printing process, leading to radiation scattering and peak broadening.^17−19^

The frequencies of the Fabry–Perot resonances can be utilized to obtain the approximate index of the material as a function of frequency^20−22^ through the well-known relation:
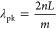
1Here *n* is the refractive index of the mesh, *L* is the thickness, and *m* is the mode order. Once the refractive index is determined, the dispersion relation can be obtained from ω = *cnk*. As shown in Figure 2c, this gives excellent agreement with the simulated dispersion of the transmitted mode. While the behavior of a wire grid metamaterial is well-established, for the simplest case of a cubic grid, there has been limited experimental evidence showing the dispersion relation across the Brillouin zone for this structure due to difficulties in fabrication,^10,11^ which we here demonstrate can be surpassed using 3D printing techniques.

## Antenna Coupling to the Double Mesh

**Figure 3:**
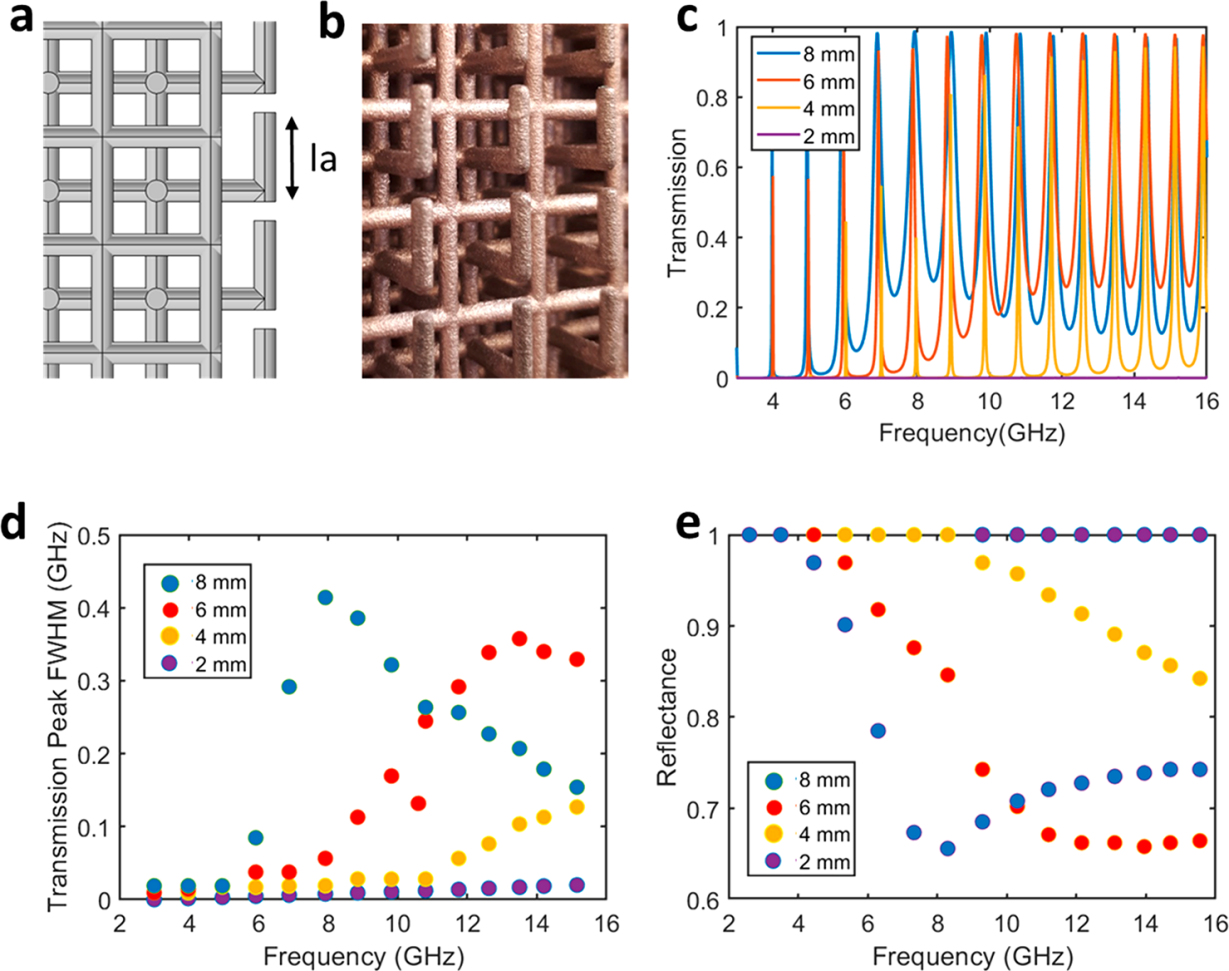
Modeling the impact of attaching monopole antennas to one grid. (a, b) Design and printed sample of the antenna structure with the monopole antennas attached to one of the nested grids. (c) Model transmission for a 1 cm grid 10 unit cells deep constructed of perfectly conducting rods terminated via different length antennas in order to explore the effect of antenna dimension on transmission. In (d), the fwhm widths of the Fabry–Perot peaks in the model transmission plot are displayed as a function of frequency, and in (e) the reflectance of the double mesh surface with antennas is shown in order to demonstrate the strength of the antenna coupling to the double mesh for different antenna lengths.

However, for the double mesh structure in Figure 1b, observing the transmission for the unaltered structure is not an option, since, as discussed, it is not possible to couple to the longitudinal “dark” mode of the structure with a plane wave. To overcome this, we added a monopole antenna at right angles to the end of each of the rods terminating at a pair of faces for *one* of the grids as shown in Figure 3. Through this design, the electric field of an incident plane wave is able to interact with the monopoles, which then couple to the longitudinal modes via a similar mechanism to an antenna at the end of a coaxial cable. At the opposite face of the structure, the reverse process occurs and the longitudinal mode causes current oscillations in the monopoles which leads the structure to radiate a plane wave.

Since the short additional antennas have a resonance defined by their length, they will only allow effective coupling to the structure around this resonance. In Figure 3c, it can be seen that while Fabry–Perot peaks exist for all frequencies where the mode of the double mesh exists, there are regions where the peaks are broad and regions where they are almost delta functions. This behavior relates directly to the antenna resonance, since with no antennas the surface of the double mesh reflects perfectly (*R* = 1) due to the mode mismatch, whereas with the addition of the antenna it can transmit radiation near the antenna resonance, so the surface reflection is reduced. The finesse, *F*, is a measure of a sharpness of the interference fringes and is directly dependent on the reflection coefficient and inversely proportional to the fwhm:
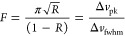
2Therefore, by measuring the peak width and peak spacing, the reflection coefficient, *R*, at the edge of the material due to the antennas can be extracted from the transmission data. This reflection coefficient is determined by the impedance of the antenna and that of the grid, and will of course vary with frequency due to the antenna resonance.^23^

**Figure 4:**
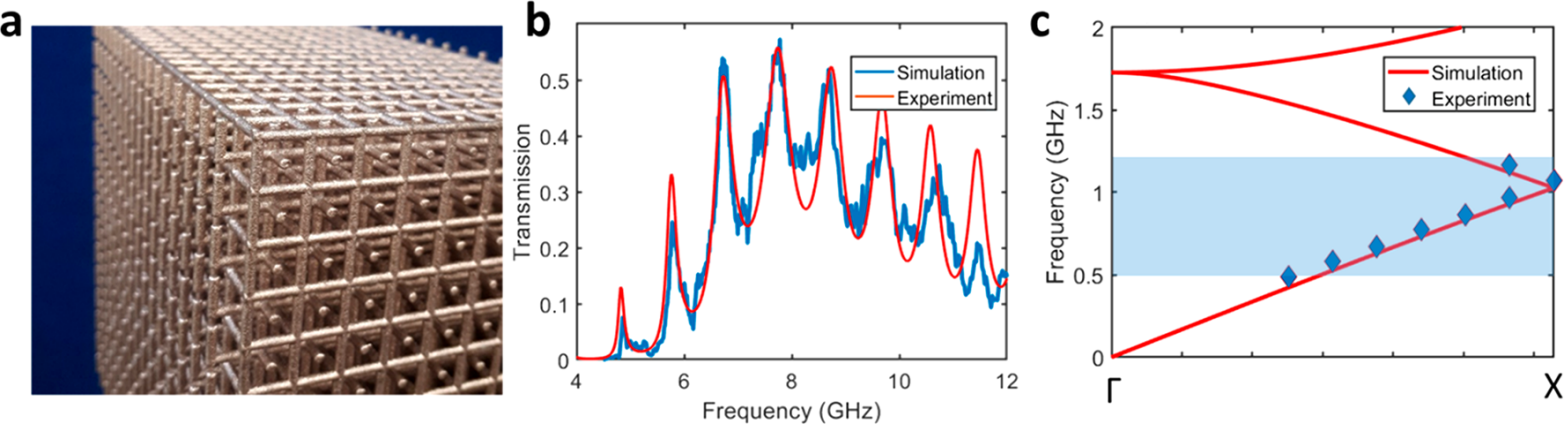
Experimental demonstration of transmission through a real sample. (a) Photograph of the metallized 3D printed double mesh. (b) Simulated and experimental transmission for a double grid with a lattice parameter of 1 cm, a wire radius of 0.9 mm and RMS surface roughness of 0.1 mm, with 8.2 mm monopole antennas and a conductivity of 1.85 × 10^5^ S/m. (c) Simulated and experimental dispersion relation of the grids, derived from the Fabry–Perot peaks. The blue shaded region denotes the fwhm of the peak for a 8 mm antenna in Figure 3d.

**Figure 5:**
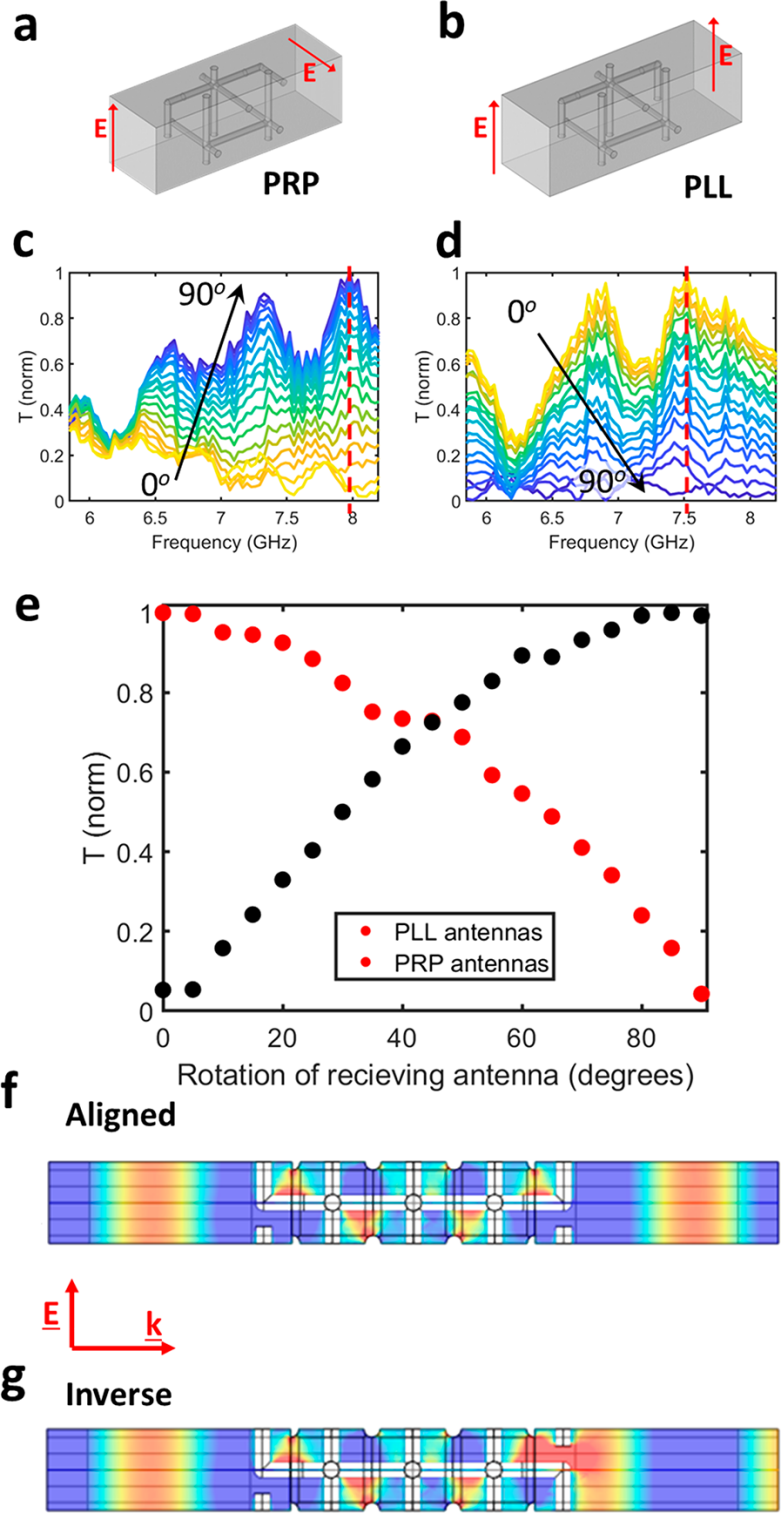
(a, b) Illustration showing a single unit cell of the double mesh with the end antennas oriented perpendicular and parallel on either end face. (c) Measured transmission corresponding to a WR 137 waveguide through a 14 unit cell mesh with the antennas at each face at 90° as the receiving horn antenna is rotated with respect to the emitting horn. (d) The same for a 10 unit cell mesh with the antennas parallel. Mesh dimensions are the same as described previously, and values are normalized to the maximum. (e) Normalized transmission values for both grids at their maximum value (dashed line in (c), (d)) as the receiving horn antenna is rotated. (f) Electric field of radiation transmitting though a double mesh with identical antennas at either end of the mesh. (g) Electric field of radiation at 11.5 GHz transmitting though a double mesh with antennas inverted at the rear face compared to the front, demonstrating a π-phase inversion for radiation passing through the structure compared to the previous case.

Therefore, the transmission plots in Figure 3c can be viewed as the FP response of a dielectric with the same effective index as the double mesh, but whose reflectance is defined by the impedance match between the antennas and the grid. By measuring the fwhm of the peaks, it is possible to extract the position of the antenna resonance (Figure 3d) and the corresponding reflectance of the structure via eq 2 (Figure 3e). As can be seen in Figure 3d and e, for longer antennas, the spectral window where *R* ≠ 1 shifts to lower frequencies. The lower limit of this is dictated by the size of the antenna, which for a 1 cm mesh with antennas parallel to one of the cubic axes cannot be larger than the unit cell without them touching and causing a short-circuit (although shown in Figure 5, the angle of the antennas with respect to the grid does not affect the field inside the structure, and so antennas at a small angle to one of the in-plane grid axes could in principle be made arbitrarily long, although in practice this would be limited by thickness of the 3D print). As we are principally interested in the nondispersive, low frequency mode of this structure, we select an 8 mm antenna to explore the broadest range of interest while still being readily fabricated. It should be noted that Figure 3c shows modeling results for the case of a perfect electrical conductor and any losses introduced into the system will rapidly degrade the narrow peaks far from the antenna resonance, making them difficult to observe in practice and the observable resonances are defined by the antenna window as seen in Figure 4.

After establishing the addition of monopole antennas to opposite faces of one of the meshes as a reliable and readily fabricable method for coupling an incident plane wave into the interlaced double mesh, a sample was fabricated using the same method as for the single mesh described earlier and is shown in Figure 4a. Once again the transmission is simulated using Comsol and measured using a pair of horn antennas connected to a VNA – as Figure 4b shows. Again the results show excellent agreement in terms of frequency, although the peaks appear broader and weaker than for the single mesh which can be attributed to the increased scattering from the surfaces owing to the larger density of wires in the grid.^17−19^

Using the same approach as for the single mesh structures, the dispersion relation for the longitudinal mode of the interlaced double mesh was determined from the transmission plots and is shown in Figure 4c. Due to the limitations imposed by the antenna lengths, a good signal was only achieved within the window given by the fwhm of the antenna resonance as taken from Figure 3d, but this is more than sufficient to verify the nondispersive, broadband nature of the longitudinal “dark” mode generated by the nested double mesh structure.

## Polarization Rotation and Phase Control

A further interesting feature of this structure is that the character of the mode within it is independent of the antennas used to excite it. Therefore, any antenna in any orientation (as long as there is an electric field component of the incident wave parallel to the antenna) can be used to excite the modes of the double mesh and likewise any antenna of any orientation can be used to couple light out. This makes the structure an efficient converter of electromagnetic energy to different polarization states, which has applications in imaging, sensing, and beam shaping.^24−26^

In Figure 5, we demonstrate the use of this structure both to achieve polarization rotation and a π phase flip. Figure 5c shows the maximum experimental normalized transmission of a double mesh with antennas on opposite faces both parallel and perpendicular to each other as the receiving horn antenna is rotated in relation to the emitting antenna, which is always aligned parallel to the antennas on the front face. It can be clearly observed that when both horn antennas are aligned to the same polarization, the transmitted signal is strongest for a double mesh with parallel antennas on opposite faces and near zero for one with antennas on the rear face rotated 90° to the antennas on the front face. When the receiving horn antenna is rotated 90° to the emitting antenna, the converse is true, demonstrating that this structure is capable of producing complete polarization rotation. As each antenna is independent of its neighbors, this principle could be used to achieve beam shaping effects such as vector beam generation.

This principle can be extended to demonstrate control of the phase exiting the double mesh, as shown in Figure 5f and g, where the antenna at the far end of a simulated double mesh structure is aligned in either an “up” or a “down” configuration. It can be clearly seen that there is a phase shift of π in the outgoing radiation between the two structures.

## Conclusions

In this study, single and double interlaced metallic meshes were fabricated using an SLS 3D printer and then metallized using conductive paint. Measurements of transmission through the structures showed good agreement with simulations, as did the dispersion relations extrapolated from experimental data. In addition, we experimentally probe the “dark” longitudinal modes of an interlaced double mesh structure by terminating one of the nested meshes with a monopole antenna, which allows free-space plane waves of frequencies about the antenna resonance to couple to the modes inside the structure. This is the first experimental demonstration of a new class of 3D metamaterial. Furthermore, we show that the longitudinal mode of the structure is independent of the orientation of the antennas used to excite it. Therefore, the structure can be used to efficiently rotate polarization or control the phase of the radiation leaving it, which has applications in imaging, sensing, and beam shaping.

## Notes

**Data Availability**. The data that support the findings of this study are available from the corresponding author upon reasonable request.
